# Enhanced Accuracy for Multiclass Mental Workload Detection Using Long Short-Term Memory for Brain–Computer Interface

**DOI:** 10.3389/fnins.2020.00584

**Published:** 2020-06-23

**Authors:** Umer Asgher, Khurram Khalil, Muhammad Jawad Khan, Riaz Ahmad, Shahid Ikramullah Butt, Yasar Ayaz, Noman Naseer, Salman Nazir

**Affiliations:** ^1^School of Mechanical and Manufacturing Engineering (SMME), National University of Sciences and Technology (NUST), Islamabad, Pakistan; ^2^Directorate of Quality Assurance and International Collaboration, National University of Sciences and Technology (NUST), Islamabad, Pakistan; ^3^National Center of Artificial Intelligence (NCAI) – NUST, Islamabad, Pakistan; ^4^Department of Mechatronics Engineering, Air University, Islamabad, Pakistan; ^5^Training and Assessment Research Group, Department of Maritime Operations, University of South-Eastern Norway, Kongsberg, Norway

**Keywords:** convolutional neural network, long short-term memory, functional near-infrared spectroscopy, mental workload, brain–computer interface, deep neural networks, deep learning

## Abstract

Cognitive workload is one of the widely invoked human factors in the areas of human–machine interaction (HMI) and neuroergonomics. The precise assessment of cognitive and mental workload (MWL) is vital and requires accurate neuroimaging to monitor and evaluate the cognitive states of the brain. In this study, we have decoded four classes of MWL using long short-term memory (LSTM) with 89.31% average accuracy for brain–computer interface (BCI). The brain activity signals are acquired using functional near-infrared spectroscopy (fNIRS) from the prefrontal cortex (PFC) region of the brain. We performed a supervised MWL experimentation with four varying MWL levels on 15 participants (both male and female) and 10 trials of each MWL per participant. Real-time four-level MWL states are assessed using fNIRS system, and initial classification is performed using three strong machine learning (ML) techniques, support vector machine (SVM), *k*-nearest neighbor (*k*-NN), and artificial neural network (ANN) with obtained average accuracies of 54.33, 54.31, and 69.36%, respectively. In this study, novel deep learning (DL) frameworks are proposed, which utilizes convolutional neural network (CNN) and LSTM with 87.45 and 89.31% average accuracies, respectively, to solve high-dimensional four-level cognitive states classification problem. Statistical analysis, *t*-test, and one-way *F*-test (ANOVA) are also performed on accuracies obtained through ML and DL algorithms. Results show that the proposed DL (LSTM and CNN) algorithms significantly improve classification performance as compared with ML (SVM, ANN, and *k*-NN) algorithms.

## Introduction

Neuroergonomics is a research field that is focused on the estimation of the brain responses generated as a result of human behavior, physiology, emotions, and cognition; in general, it is the study of human brain and its behavior at work (Mehta and Parasuraman, 2013; Curtin and Ayaz, 2018; Ayaz and Dehais, 2019). Passive brain–computer interface (pBCI) is one of the important research areas of neuroergonomics. pBCI is designed using the arbitrary brain signals to decode user intentions (Khan and Hong, 2015). These signals may be decoded from fatigue, mental workload (MWL), drowsiness, vigilance, stress, anxiety, and so forth. The passive brain activities are decoded for monitoring applications to ensure a reliable decision-making process. Among these passive brain activities, MWL is a complex function that involves neurophysiologic processes, perception, short-term memory (STM), long-term memory (LTM), and cognitive functions (Bergasa et al., 2018). Exceeded limits of MWL are mostly the cause for irrational decision making that can lead to errors and safety hazards (Byrne et al., 2013). Drowsiness, one of the passive brain activities, is a major cause of traffic accidents (Bioulac et al., 2017). In the present realm of human–machine interaction (HMI), modern technology requires even greater cognitive demands from users and operators for ensuring safety and maximizing the effectiveness (Saadati et al., 2019a).

There are different approaches toward estimation of MWL: subjective rating, performance, and physiological measures are the most common techniques. The performance rating method keeps track of a person’s progress by using two metrics, namely, accuracy (a person’s deviation from fixed procedure) and reaction time (how fast task is done), whereas the subjective rating methods use questioners that are designed by evaluators to assess the emotional and cognitive states of the subject. Also, self-reporting and opinions of the subjects during the experimentation are also considered to measure the MWL (Chen et al., 2019). Several research studies use tests like National Aeronautics and Space Administration’s Task Load Index (NASA-TLX) and subjective workload assessment technique (SWAT) to measure the cognitive load (Noyes and Bruneau, 2007). A limitation of subjective method is the self-reporting protocol that is dependent on the respondent’s opinion, which may be affected by self-feelings, biasedness, low motivation, ambivalence, and mistakes in interpreting environment changes (Paulhus and Vazire, 2007). In addition, these methods may not consider the physical work associated with the activities involving movement of a person’s arms, legs, feet, or entire body (Cain, 2007). On the other hand, physiological methods provide a real-time assessment and higher feasibility. The physiological techniques also require a smaller sample size to estimate reliable cognitive load states (Tran et al., 2007). Physiological sensors, such as electroencephalogram (EEG), heart rate variability (HRV), eye response measurement, functional magnetic resonance imaging (fMRI), and functional near-infrared spectroscopy (fNIRS) are most commonly used for the monitoring of the MWL (Hong and Santosa, 2013; Tong et al., 2016; Curtin et al., 2019).

Electroencephalogram is commonly used modality for monitoring passive brain activities (Frey et al., 2014). In the domain of functional neuroimaging, EEG has certain robust advantages over the other techniques (J. Ph Lachaux et al., 2003; Harrison and Connolly, 2013; Wang et al., 2019) and used extensively in cognitive neuroscience and BCI applications. However, EEG has some limitations owing to its low spatial resolution and is usually constrained to measure the region-specific brain activities (Strait and Scheutz, 2014). fMRI does offer higher spatial resolution, but it limits the subject’s portability and struggles in temporal resolution (Canning and Scheutz, 2013). fNIRS, on the other hand, offers balanced spatial and temporal resolution as compared with other neurophysiological modalities and is widely used for MWL estimation (İşbilir et al., 2019). fNIRS systems are described in comparison with other modalities and used as a compromise between fMRI and EEG in relation to spatial and temporal resolution, respectively. Portability requirement of fNIRS system is primarily for its use in neuroergonomic applications (MWL) in ecological environment. fNIRS is also less prone to electro-psychological artifacts, easy to wear, portable, and lightweight (Naseer and Hong, 2015; Hong and Khan, 2017). Several recent studies have used fNIRS for classification of cognitive tasks and events (Abibullaev and Jinung, 2012; Ayaz et al., 2018; Asgher et al., 2019; Wang et al., 2019). These studies include motor imagery, mental arithmetic (MA), MWL, vigilance, and motor execution-based paradigms, which have been experimentally performed to measure accuracies of system. In these studies, the most important objective is to improve classification accuracies, which lead to the exploitation of appropriate classifiers using different machine learning (ML) techniques. The challenging part in these conventional ML classification methods is feature engineering, involving feature extraction, a large number of possible features, feature selection, their combinations, and dimensionality reduction from a relatively small amount of data, which leads to overfitting and biasness (Trakoolwilaiwan et al., 2017; Wang et al., 2019). These intrinsic limitations make researchers tweak around and hence results in a lot of time consumed in data mining and preprocessing. Deep learning (DL) with deep neural networks (DNNs) has emerged as an alternative to overcome this challenge by bypassing the need for manual feature engineering, data cleaning, transformation, and reduction before feeding into learning machines (Saadati et al., 2019a).

Linear discriminant analysis (LDA), *k*-nearest neighbor (*k*-NN), and support vector machine (SVM) have been rigorously implemented and are well-studied classification algorithms in BCI and MWL analyses (Tai and Chau, 2009; Power et al., 2012; Hortal et al., 2013; Naseer and Hong, 2013; Sumantri et al., 2019). All these conventional classifiers and ML algorithms are hampered by complex feature engineering and dimensionality reduction in order to make data visible to the learning system. DNNs have recently gained popularity as highly efficient training classifiers, but limited studies are available so far (Zhang et al., 2019; Saadati et al., 2019b). Hennrich et al. (2015) and Naseer et al. (2016) used DNN and other conventional classifiers to differentiate between two and three cognitive states using brain fNIRS signals. Some studies used similar procedures for binary classification to control robot and gender classification (Ozge Mercanoglu et al., 2017; Huve et al., 2018). Saadati et al. (2019a, b) employed CNN with hybrid fNIRS–EEG for MWL classification and neurofeedback. Various studies (Abibullaev et al., 2011; Ho et al., 2019) modeled deep belief network (DBN) and CNN framework for discriminating MWL and left and right motor imagery tasks using multichannel fNIRS signals. Long short-term memory (LSTM) is one of the variants of DL–recurrent neural network (RNN) algorithms specifically designed for time-series data (Schmidhuber and Hochreiter, 1997). The only available work on LSTM is that of Yoo et al. (2018), which is limited to only three class classifications and has not compared LSTM results with most employed CNN algorithms.

**FIGURE 1:**
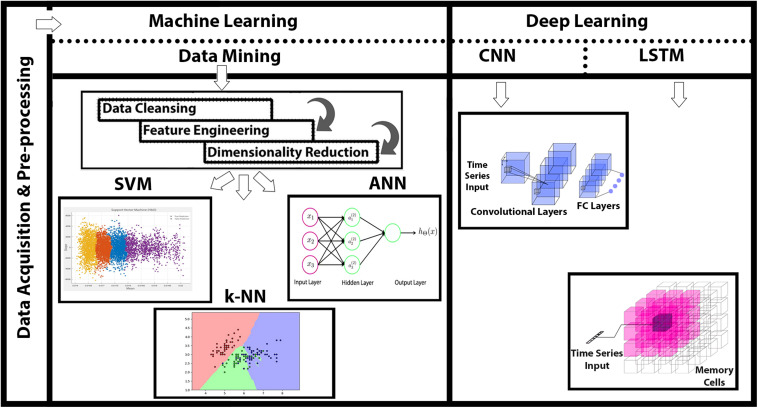
Functional near-infrared spectroscopy (fNIRS)-based mental workload (MWL) classification using machine learning (ML) and deep learning (DL) algorithms. Data acquisition through fNIRS system (P-fNIRSSyst), data preprocessing, and detailed feature engineering followed with application of ML and DL classification.

In this study, we acquired a four-level MWL with varying difficulty levels using fNIRS from 15 healthy subjects (including both male and female). Physiological noises and other high-frequency artifacts were removed using low-frequency bandpass (fourth-order Butterworth) filter (Santosa et al., 2013). Statistical significance of data is verified by *p*- and *t*-tests. Three ML classifiers [SVM, *k*-NN, and artificial neural network (ANN)] along with two DNN algorithms (CNN and LSTM) are used in the analysis and classification of four-state MWLs. The major contribution of this research is that, for the first time, LSTM is applied directly on four-class MWL-fNIRS sequential data for classification and comparison with CNN. ML classifiers couldn’t perform well in comparison with DNN algorithms; and within the DL paradigm, the LSTM offers significantly better classification accuracy than does the CNN. The comprehensive summary of research is depicted in Figure 1.

## Methods and Methodology

### Experimental Protocol and Experimentation

#### Methodology

**FIGURE 2:**
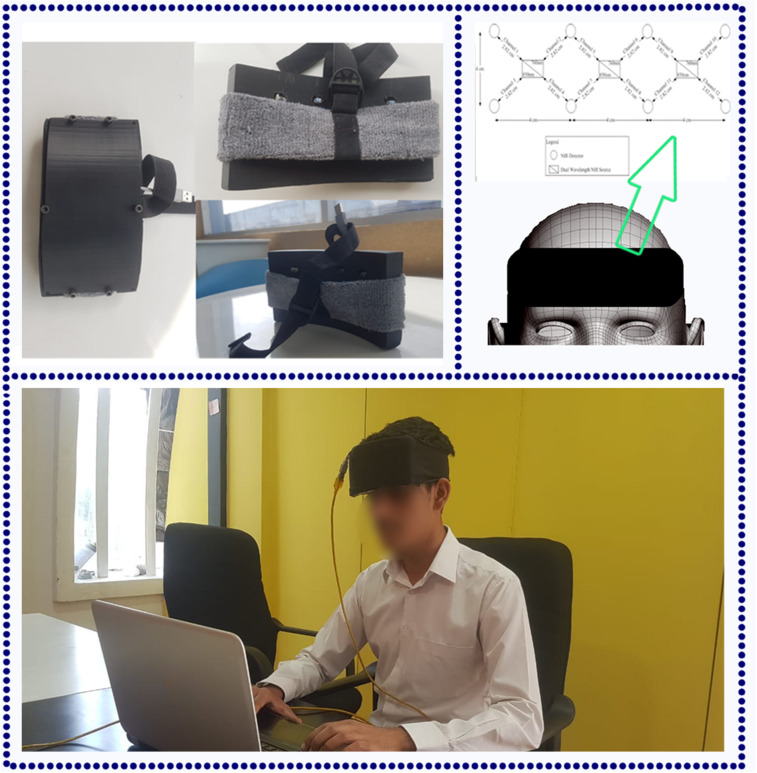
The functional near-infrared spectroscopy (fNIRS) (P-fNIRSSyst) system placed to measure participants’ prefrontal cortex (PFC) activity. Optodes are placed according to the standard 10–20 system.

In this study, 12 channels [12 oxyhemoglobin (HbO) and 12 deoxyhemoglobin (HbR)] and two-wavelength (760 and 850 nm) continuous-wave fNIRS system, namely, “P-fNIRSSyst” is used to measure neuronal activity in form of hemodynamic concentration changes in prefrontal cortex (PFC) (Asgher et al., 2019). There is a 20-ms delay between reading channels and triggering light source, and 3 μs is employed to obtain voltage values of channels. Data samples are acquired at a rate of 8 Hz (per channel per second), which effectively translates into 192 samples per second [12 channels × 2 (both HbO and HbR) × 8 (per channel sample rate) = 192]. fNIRS optical optodes are placed in an arrangement as shown in Figure 2.

#### Experimental Conditions and Participants

Ten male and five female subjects (all right-handed; age range of 20–27 years, with a mean age 23.5 years and standard deviation of 5.5 years) participated in this experiment; they also have an educational background in engineering and technology. Before the final selection, a medical screening test is conducted with the supervision of a medical physician. None of the subjects had any mental, visual, or psychological disorder. Participants are given the details and procedures of the experiment prior to the start of the experiment. All the experiments are conducted in accordance with the Declaration of Helsinki and are approved by the Ethical Research Council of RISE at SMME—National University of Sciences and Technology (NUST). The task environment is designed such that minimum external interference and artifacts should entail in readings. The dark and quiet room is selected with a comfortable back support chair to ensure restful experience (Hong et al., 2015). After an initial relaxation period, participants are asked to put on the fNIRS forehead band on the scalp as shown in Figure 2 and sit in front of the laptop screen. It is a supervised experiment; participants are observed with a live stream video camera placed in front of them from an adjacent room.

### Data Acquisition

#### Experimental Tasks and Paradigm

**FIGURE 3:**
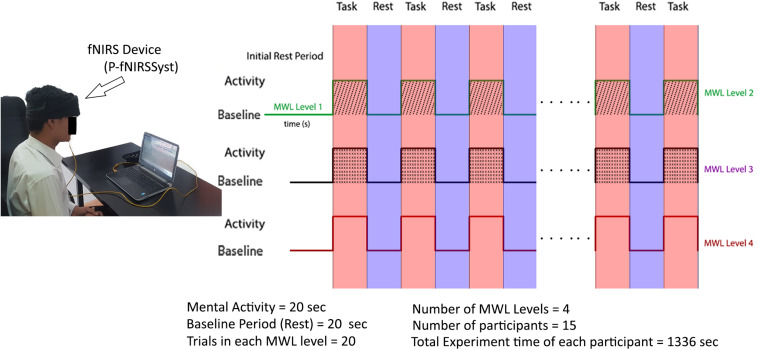
Data acquisition and experimental paradigm with experimental trials and trial sequence diagram. After initial rest period, participants are presented through mental workload (MWL) tasks in phases with increasing difficulty order.

The experiment is designed to discriminate between four levels of MWL. The participants are asked to restrict their physical and head movements as much as possible in order to avoid the artifacts. At the start of the experiment, participants are presented with Microsoft Office PowerPoint (version 16.0) slides shown on the laptop screen placed at 70 cm from nasion. The MA task is selected to evoke the brain activity and to entail a certain amount of MWL, which is prominent in case of MA problems (Power et al., 2011, 2012; Schudlo and Chau, 2013; Kosti et al., 2018). Here, the objective is to measure the mental cognition on the basis of the logic and arithmetic and to ascertain different brain activities with different difficulty levels and their classification. The participants were required to complete the task in time with accuracy. In order to set a baseline, an initial 146 (120 + 26) s are given as a rest period to settle all the brain signals at baseline. The baseline is followed by 20-s MA activity task to gauge MWL level 1 (MWL-1); next, 20 s is the relax (rest) period of the brain, and the brain attains baseline reference during the rest period (during the rest period, the participants are asked not to focus at any point). The first MWL-1 task is designed such that it induces a minimal amount of MWL (Galy et al., 2012). Task 1 contains a simple three-number addition such as 769 + 292 and 345 + 229 as MWL-1. Similarly, in phase I, the participants are again given consecutive second tasks to gauge MWL-1 for the same period of 20 s followed by a rest period of 20 s. During the rest period, the participants were asked to relax their mind and place mind at rest (Power et al., 2012; Naseer and Hong, 2013; Schudlo and Chau, 2013), so that no brain activity is generated during rest, whereas focusing on a point or cross in turns could generate a brain activity (Izzetoglu et al., 2011), which was not required in this study, and that could be easily differentiated from the mental math task. This pattern is repeated 10 times, with 10 trials for each participant consisting of MWL-1. After completion of MWL-1, the participants are presented with workload level 2 task in similar conditions. MWL level 2 (MWL-2) starts with a 25-s baseline (rest) period after the MWL-1 and followed the same pattern of 10 trials of MA task 2 (MWL-2) with the 20-s duration of each trail and 20-s rest period in between each MA task. MA task-2 is designed such that it creates a moderate amount of MWL-2 (Galy et al., 2012; Longo, 2018) in a fixed time window. The difficulty level is MWL-2 > MWL-1. MWL-2 has slight complex calculations as compared with MWL-1, including addition, subtraction of large numbers, and operations like multiplication and division, for example, 692 - 579, 60 × 11, and 49/29. Similarly, MWL-3 starts after a rest period of 25 s and has complex MA tasks to induce a high level of MWL. The difficulty level is MWL-3 > MWL-2. It includes arithmetic operation on equation, and the resultant answer (ANS) is utilized in the next calculations (e.g., 823 - 3, ANS × 3, ANS - 21, and ANS + 211) involving mental math task, mental logic, and memory element (Herff et al., 2013; Hosseini et al., 2018). fNIRS recording activity for MWL-1 took 546 s, MWL-2 took 405 s, and MWL-3 took also 405 s for each participant. The total time of experiment of 10 trials with the three MWLs and rest is (546 + 405 + 405) = 1336 × 15 = 2040 s. The tasks timeline sequence of three MWL levels and rest period (four cognitive states) is shown in Figure 3. Experimental tasks are verified using standard subjective assessment measure NASA-TLX method. Here, class is an activity (category) having a specific cognitive difficulty level (MWL), which is categorized from other classes (MWL levels) or categories using ML and DL classification. The NASA-TLX is a subjective, multidimensional assessment tool that rates perceived MWL in order to assess task, gauge cognitive workload, effectiveness, and performance. Experimental paradigm is repeated, and questionnaires are filled with subjects’ input. Results show the reliability of experimental tasks and the difficulty levels (classes) of various MWLs. The TLX (index) weight is MWL-3 > MWL-2 > MWL-1. The results obtained using NASA-TLX are shown in Annexure A (Supplementary Material) that validate the experimental paradigm for MWL assessment and analysis.

#### Data Preprocessing

Brain activity is detected by measuring concentration changes of HbO and HbR molecules in the microvessels in the cortex. The modified Beer–Lambert law (MBLL) and its variants FV-MBLL are used for measuring concentration changes of HbO and HbR using the information on the intensities of detected NIR light at two different time instants (Pucci et al., 2010; Asgher et al., 2019).

(1) $$\left[\begin{array}{c} \Delta C_{\text{HbO}}\left(t_{i}\right)\\ \Delta C_{\text{HbR}}\left(t_{i}\right) \end{array}\right]=\frac{{\left[\begin{array}{c} \alpha_{\text{HbO}}\left(\lambda_{1}\right)\;\alpha_{\text{HbR}}\left(\lambda_{1}\right)\\ \alpha_{\text{HbO}}\left(\lambda_{2}\right)\;\alpha_{\text{HbR}}\left(\lambda_{2}\right) \end{array}\right]}^{-1}\left[\begin{array}{c} \Delta OD\left(t_{i};\lambda_{2}\right)\\ \Delta OD\left(t_{i};\lambda_{2}\right) \end{array}\right]}{l\times xd}$$

Detected raw voltage readings from fNIRS optodes of detected lights are processed through analog-to-digital converter (ADC) and are sent to the computer through Bluetooth connection, where they are normalized by dividing with the mean value. Then signals are passed through low-passed band filter using a fourth order, with zero-phase Butterworth filter (Naseer et al., 2014) having a cutoff frequency of 0.3 Hz to remove high-frequency artifacts due to breathing (0.2–0.5 Hz), blood pressure (∼0.1 Hz), and heartbeat (1–1.5 Hz) (Franceschini et al., 2006; Huppert et al., 2009). Then relative hemodynamic concentration changes are calculated according to Naseer and Hong (2013). The time-series waveforms for different MA (MWL) tasks and MWLs are easily segregated and plotted and are included in Annexure B (Supplementary Material). Here, the response activities show different difficulty levels of MWL and can be easily segregated and classified as time-series data.

#### Statistical Significance of Functional Near-Infrared Spectroscopy Data

**FIGURE 4:**
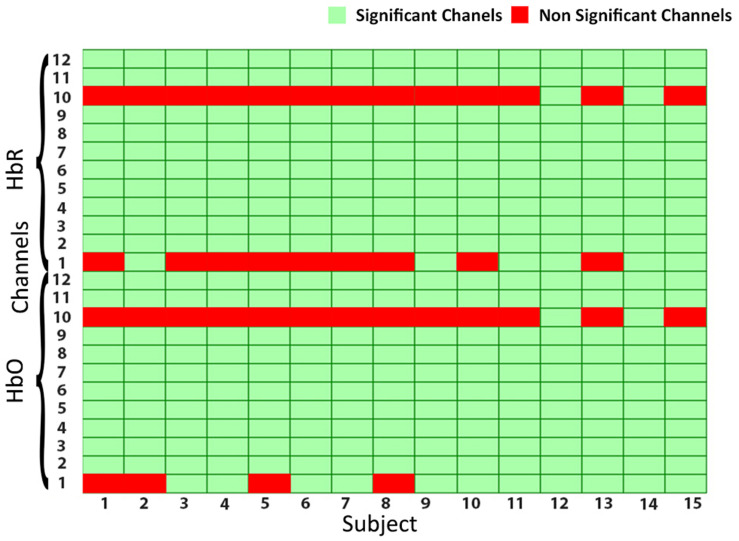
The statistical significance of channels: green cells showing the significant channels, whereas red cells are non-significant channels.

Functional near-infrared spectroscopy optodes are placed on the forehead (PFC) of subjects as shown in Figure 2. Amplitude and intensities of acquired hemodynamic signals vary from person to person and depend on various factors (Hong and Khan, 2017). The data validation and function response of the device is mentioned in Asgher et al. (2019). Further, in order to determine the integrity and validity of four-class data acquired from the fNIRS system and to make sure that each channel of the device has significant information, a statistical significance of data per channel is first calculated. Independent-samples *t-*test and *p*-test are calculated with the null hypothesis: There is no significant difference between collected fNIRS data and standard data patterns and alternate hypothesis as otherwise on each channel. Additional parameters are also considered like negative correlation between HbO and HbR and channel data comparison with MWL model. For channels having a *p*-value of less than 0.05, we rejected the null hypothesis and accepted the alternate hypothesis. For all subjects, data from only those channels that fulfill the criteria are considered, as given in Figure 4. The figure shows the data significance per channel. Green bars in the figure show that 89.16% of the acquired data are significant.

## Data Mining and Feature Engineering

**FIGURE 5:**
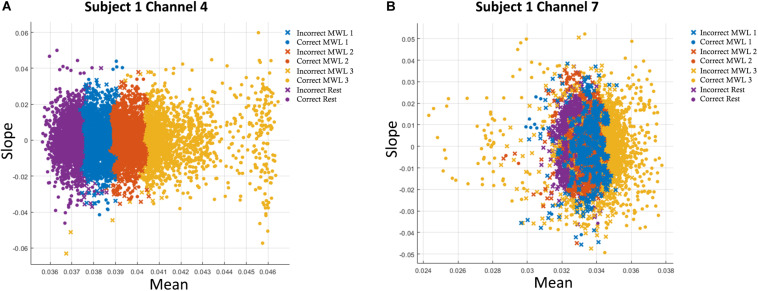
**(A)** Accuracies with two-feature (mean–slope) combination in support vector machine (SVM) classification plot of oxyhemoglobin (HbO) for subject 1 on channel 4. **(B)** Accuracies with two-feature (mean–slope) combination in *k*-nearest neighbor (*k*-NN) classification plot of HbO for subject 1 on channel 7. Only statistically significant channels are considered.

After the data are preprocessed and noise is removed after filtering, the features are extracted from it for classification and discrimination. Features are directly extracted from NIR intensity signals (Power et al., 2012), and the common practice is to extract features directly from acquired hemodynamic signals (HbO and HbR) in the form of changes in concentration (ΔHbO and ΔHbR) (Santosa et al., 2017; F. Wang et al., 2018) to provide improved data cleaning and feature selection. Hemodynamic activity data of the brain can be represented in various feature forms (Bashashati et al., 2007; Lotte, 2014), and different feature combinations can be effectively used for signal classification. All extracted features are normalized in the range [0, 1] before classification. Features are selected such that they have more data information and do comprise significant information that is subsequently used for precise classification (Naseer et al., 2016). The analysis and results of ML algorithms are calculated from various feature combinations: signal mean, maxima, variance, minima, slope, variance, skewness, kurtosis, and signal peak. The obtained results show slope and mean yield the best result in our study, and the best features are mentioned in Figure 5. These feature engineering results and findings are in line with previous studies (Abibullaev and Jinung, 2012; Khan and Hong, 2015; Naseer et al., 2016; Hong and Khan, 2017).

### Feature Extraction and Selection

Selecting appropriate features for classification is vital, and most of the studies are confined to extracting optimum statistical values of hemodynamic signals. Acquiring the highest accuracy of classification depends on the number of factors such as length of the sliding window (Hong et al., 2015), choosing the best set of feature combinations (Naseer et al., 2016), wavelet functions for decomposition, and temporal and spatial resolution of modalities (Abibullaev and Jinung, 2012). After the best feature extraction techniques are mentioned, optimal features used for classification are signal mean, slope, variance, skewness, kurtosis, and signal peak (Khan and Hong, 2015; Hong and Khan, 2017). Before features are calculated, all channels were normalized between [0, 1] using the following equation:

(2) $$x_{\text{norm}}=\frac{x-x_{\text{min}}}{x{\text{min}}_{\text{max}}}$$

where *x*_norm_ is the normalized feature value between 0 and 1, and *x*_min_ and *x*_max_ are the smallest and largest values, respectively. To avoid the model’s overfitting on training data and validating classification performance, 10-fold cross-validation is used. In 10-fold cross-validation, data are divided into 10 subsets, and one subset is used as test set while the other nine sets are used as training sets, whereas in leave-one-out cross-validation (LOOCV) is logical extreme of *k*-fold cross-validation, with *k* equal to number of total data points (*N*). For a smaller dataset, LOOCV is considered suitable, whereas for medium datasets, *k*-fold cross-validation is preferred. LOOCV is also expensive in terms of computational cost and train test time. To save the computational resources and the nature of datasets lies in the medium category; therefore, *k*-fold cross-validation is employed in this study (Wong, 2015).

## Analysis and Classification Using Machine Learning Algorithms

### Support Vector Machines and *k*-Nearest Neighbor Classification

Support vector machine is the most commonly used discriminative classifier in various studies for classification and pattern recognition (Thanh et al., 2013; Khan et al., 2018). In supervised learning, given a set of labeled training data, SVM outputs an optimal hyperplane that assigns new test data to one of the categories of the classification. SVM is designed such that it maximizes the distance between the closest training points and separating hyperplanes. In two dimensions, separating hyperplane feature space is given by:

(3) f(x)=r⋅x+b

where *b* is a scaling factor and *r*, *x* ∈ *R*^2^. The loss function of SVM for a two-class classification problem is given in Eq. 4. For more than two classes, the one-versus-all approach is used in which class 1 is the class that we want to predict and all other classes are considered as class 2 using the same formula:

(4) $$J\left(\theta \right)=\sum_{i=1}^{m}y^{\left(i\right)}Cost_{1}(\theta^{T}\left(x^{\left(i\right)}\right)+\left(1-y^{\left(i\right)}\right)Cost_{0}(\theta^{T}\left(x^{\left(i\right)}\right)$$

In Eq. 4, *m* represents the total number of data points. And the cost is calculated as

(5) $$Cost\left(h_{\theta }\left(x\right),y\right)=\{{}_{\max\left(0,1+\theta^{T}x\right)\;\mathrm{if}y=0}^{\max\left(0,1-\theta^{T}x\right)\;\mathrm{if}y=1}$$

The common practice to do multiclass classification with SVMs is to employ a one-versus-all classifier and predict the class with the highest margin (Manning et al., 2008).

*k*-Nearest neighbor is a non-parametric method, commonly used for pattern recognition, classification, and regression tasks (Sumantri et al., 2019). In the case of the classification, the output is class label assigned to the object depending on the most common class among its *k*-NNs. Weights are assigned to the test point in inverse relation to the distance, that is, 1/*D*, where *D* is the distance to the neighbor, such that neighbors near the input are assigned more weight as the distance is less and vice versa as given in Eq. 6.

(6) $$D\left(x,p\right)=\left\{\begin{array}{c} \sqrt{{\left(x-p\right)}^{2}}\;\mathrm{Euclidean}\\ {\left(x-p\right)}^{2}\;\mathrm{Euclidean}\mathrm{eSquared}\\ \left(x-p\right)\;\mathrm{Manhattan} \end{array}\right\}$$

Training dataset in case of *k*-NN are vectors in multidimensional feature space with each class label. In the prediction phase of the algorithm, the distance of an unlabeled input is calculated using Euclidean distance. Data are in pairs like (*x*_1_,*y*_1_),(*x*_2_,*y*_2_),….(*x*_*n*_,*y*_*n*_) ∈ ℝ^*d*^ such that ℝ^*d*^*x*{1,2} where x is the feature, y is the class label of the feature, and *p* is the query points. Predictions are made on the basis of *k*-NN examples by using formula (7) (Zhang et al., 2018).

(7) $$y=\frac{1}{k}\sum_{i=1}^{k}y_{i}$$

*k* is a hyperparameter and its selection depends on the data. Generally, larger values of *k* reduce the effects of noise on the classification but make boundaries less distance between the classes. Here, SVM and *k*-NN are implemented to discriminate between four MWL levels from fNIRS datasets of 15 participants.

All algorithms were trained and tested on MSI GE62VR Apache Pro Laptop with NVIDIA GEFORCE^®^ GTX 1060 having a 3 GB GDDR5 graphic card. SVM and *k*-NN were performed on MATLAB 2019a Machine Learning app, whereas ANN, CNN, and LSTM were performed on Python 3.7 on Anaconda SPYDER integrated development environment (IDE). In both ML and DL algorithms, Adam optimizer is used to dynamically adjust the learning rate and is the most (Kingma and Ba, 2015). At the start of training, the weights are initialized from Xavier uniform distribution (Glorot and Bengio, 2010). For SVM and *k*-NN, we extracted nine features from the original hemodynamic HbO and HbR signals, namely, mean, median, standard deviation, variance, minima, maxima, slop, kurtosis, and skewness. These features were spatially calculated across all 12 channels with a moving overlapping window of 2 s. For two feature combinations, Signal Mean (M) and Signal Slope (S) produced the best results, which are shown in Figures 5A,B for Subject 1. Average accuracies across 12 channels show that average classification accuracy achieved with SVM and *k*-NN is 54.33 and 54.31%, respectively.

### Artificial Neural Network Classification

An ANN has at least three layers (an input layer, a hidden layer, and an output layer), and each layer performs its computation and learning tasks, where the number of neurons in each layer depends on the number of inputs in the input layer and the number of outputs in the output layer. The hyperparameters are neurons in each layer, weights, network structure, and learning parameters that are learned by training the network again and again to get the maximum accuracy.

The output of a neuron is mathematically expressed as

(8) $$a_{i}^{\left(j\right)}=g\left(\theta^{\left(j\right)}x_{k}\right)$$

where *a*_*i*_^(^*^*j*^*^)^ is the activation of unit *i* in a layer and *g* is the activation function applied, θ^(^*^*j*^*^)^ is the matrix of weights controlling function mapping from layer *j* to layer *j* + 1, and *x*_*k*_ is the input from the previous layer of neurons or initial input. The recursive chain rule is implemented to calculate gradients during backpropagation. Mathematically, the chain rule is defined in Eq. 9. The cost function for ANN is given in Eq. 10.

(9) $$\frac{dy}{dx}=\frac{dy}{du}.\frac{du}{dy}$$

$$J\left(\Theta \right)=-\frac{1}{m}[\sum_{i=1}^{m}\sum_{k=1}^{K}y_{k}^{\left(i\right)}\log h_{\theta }{\left(x^{\left(i\right)}\right)}_{k}+$$

(10) $$\left(1-y_{k}^{\left(i\right)}\right)\log\left(1-h_{\theta }{\left(x^{\left(i\right)}\right)}_{k}\right)]+\frac{\lambda }{2m}\sum {}_{l=1}{}^{L-1}\sum {}_{i=1}{}^{\delta_{l}}\sum {}_{j=1}{}^{\delta_{l+1}}(\Theta_{j}^{\left(l\right)}){}^{2}$$

**FIGURE 6:**
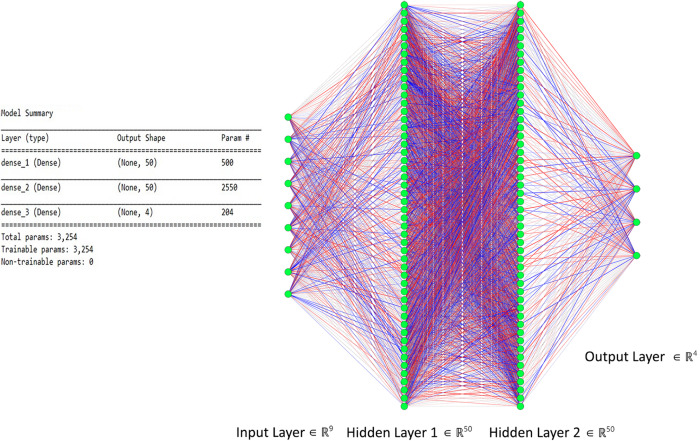
The proposed artificial neural network (ANN) model with two hidden layers used for classification.

The proposed ANN model consists of two hidden layers along with input and output layers. The dimension of the input layer corresponds to selected features, whereas the output layer corresponds to distinguishable MWL classes, which, in our case, are 9 and 4, respectively. Each hidden layer consists of 50 neurons and is fully connected with the previous and next layers. For activation function in hidden layers, “Relu” is used, which introduces non-linearity to learn complex features, given in Eq. 11. The output layer has a “sigmoid” activation function for multiclass classification and prediction. The ANN model summary used in this study is shown in Figure 6 with details about layers, neurons, and parameters used in this study. Every channel for each subject with nine extracted features is passed through the network, and cost is calculated through gradient descent. Loss is backpropagated through network, and weights are adjusted. This process is repeated equally to the number of epochs, that is, 100. At each epoch, accuracy is calculated; later, the accuracy is averaged out on all 12 channels and is segregated and will be discussed in section “Results”.

(11) $$f\left(x\right)=\left(x^{+}\right)=\text{max}\left(0,x\right)$$

## Analysis With Deep Learning Classification Techniques

### Convolutional Neural Networks

Convolutional neural networks (CNNs) intelligently adapt the inherent properties of data by performing different operations on the data as a whole and extracting key feature before feeding it into fully connected layers (LeCun and Bengio, 1995; Dos Santos and Gatti, 2014). The acquired fNIRS dataset has specific patterns within it, which relates to the strength of mental activity with hemodynamic concentration changes (ΔHbO and ΔHbR). CNN has to learn this hidden pattern on its own (without human intervention, i.e., manual feature engineering) through end-to-end training (Ho et al., 2019; Saadati et al., 2019a). CNNs have one, two, or multiple convolutional layers with an activation function along with pooling layers to adjust the dimensions of the feed data, but these layers are not fully connected. Resultant layers formed after convolution operation are known as activation maps. These activation maps hold the features and patterns within fNIRS training data required for successful classification. The number of filters must be the same as the input data depth to perform convolution, and the output size of the resulting activation map is determined by the filter size and stride using the following formula.

(12) $$\mathrm{Output}\mathrm{size}\left(W,H\right)=\frac{\left(\text{N-F}\right)}{\text{stride}}+1$$

where *N* is the dimension of input data; *F* is dimension of filter; and Stride is the step length for convolution. Convolution of the input signal and filter weights is performed as a convolution of two signals, that is, element-wise multiplication and sum of a filter and the signal (i.e., time-series fNIRS data).

(13) f[x,y]g*[x,y]=∑n1=-∝∝∑n2=-∝∝f[n1,n2]⋅g[x-n1,y-n2]

**FIGURE 7:**
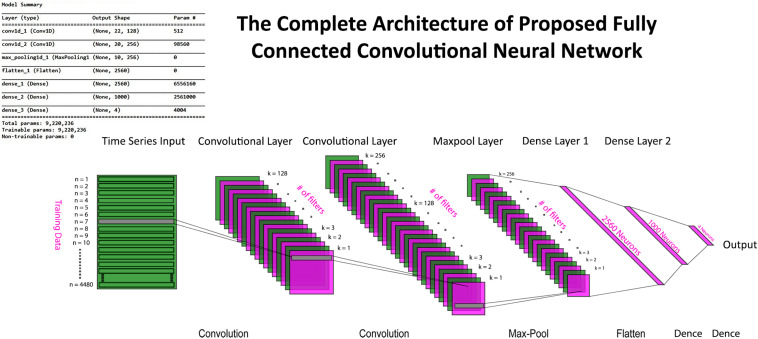
The complete convolutional neural network (CNN) model with input, convolution, max pool, dense, and output layers. Model summary include details about hyperparameters and network architecture.

**FIGURE 8:**
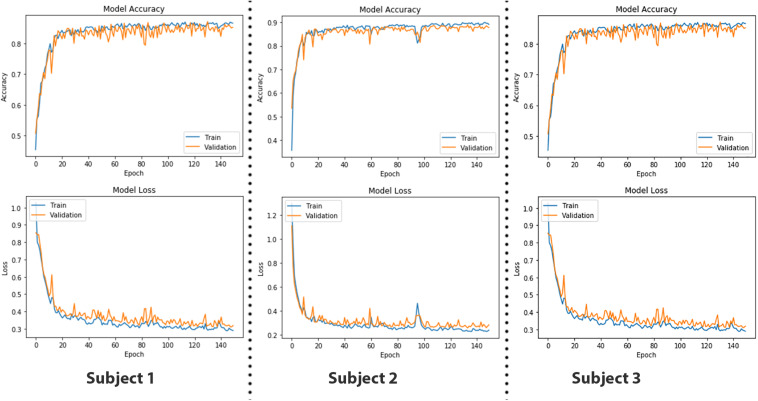
The epoch versus accuracy and loss plots of convolutional neural network (CNN) model on train and validation datasets. Accuracy increases with number of epochs and then saturates; vice versa for the loss.

The next important layer in the convolutional network is the pooling layer. It reduces the spatial size of the activation maps generated by the convolution operation of filters on a 12-channel data stream. The output size of volume produced as a result of pooling is determined by

(14) $$\mathrm{Output}\mathrm{size}\left(W,H\right)=\frac{\left(\text{N-F}\right)}{\text{stride}}+1$$

where the depth of data remains the same, whereas width and height are reduced to half in case of max pooling with a stride having a value of 2. Input data after passing through a series of convolution and pooling layers are flattened and fed into the fully connected layers to perform the classification task. The complete parameters and structure of the proposed CNN are shown in Figure 7. It is a fully connected feed-forward network with two convolution layers followed by one max-pooling layer, and then the output from the max-pool layer is flattened and fed into a dense layer that terminates into the final output layer before passing through another fully connected layer. There are 24 readings vector (12 HbO + 12 HbR), which served in the “Conv1D” convolutional layer. One hundred twenty-eight filters spatially convolve with the input data stream and learn high-level features for classification in the form of activation maps. Figure 8 represents the graphs of accuracy and loss over train and validation sessions on different subjects. A batch size of 500 is used to train the network over 150 epochs.

### Long Short-Term Memory

**FIGURE 9:**
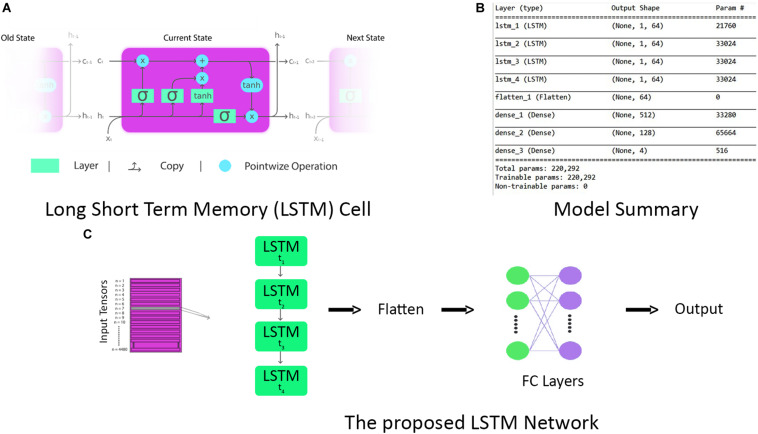
**(A)** The repeating long short-term memory (LSTM) cell with input, forget, and output gates. **(B)** Complete model summary of the proposed LSTM network. **(C)** The architecture of the proposed LSTM network.

Long short-term memory is a modification of the RNN with a feedback connection (Schmidhuber and Hochreiter, 1997). LSTM networks are well suited for time-series data classification, processing, and predictions owing to unknown time duration lag between important events in a time series. LSTM provides better classification and learning results than do conventional CNN and vanilla RNNs (Graves et al., 2009, 2013). An LSTM unit is a cell with three gates, that is, an input gate, an output gate, and a forget gate (Greff et al., 2016), as shown in Figure 9A. The three gates regulate the flow of information in and out of the cell, enabling it to remember values over random time intervals. The cell keeps track of the interdependencies of elements in the input sequence. Often, logistic sigmoid function is used as an activation function of LSTM gates (Gers and Schmidhuber, 2001; Gers et al., 2003). Logistic sigmoid function is given by

(15) $$f\left(x\right)=\frac{1}{1+e^{-k\left(x-x_{0}\right)}}$$

where *e* is the natural logarithm base, *x*_0_ is the *x*-value of the sigmoid midpoint, and *k* is the logistic growth rate. There are connections between input and output gates of LSTM, usually recurrent. The weights of these connections are learned during the training to determine the operation of these gates.

The major takeaway of this study is the application of LSTM for the first time in the classification of a multiobjective task problem. First of all, data of each subject are split into train and validation sets with a 70:30 ratio. To make input data compatible with LSTM, they are reshaped such that for each time instance, we have a data stream of all 12 channels in a single row vector format of 24 units (12 HbO + 12 HbR). After initial preprocessing, time-series data are fed into the LSTM unit as vectors, labeled as lowercase variables in the following equations, with the matrices in uppercase variables. The equations for forward pass of LSTM unit with a forget gate are given below:

(16) $$f_{\text{t}}=\sigma_{\text{th}}\left(W_{\text{f}}x_{t}++U_{\text{f}}h_{t-1}+b_{\text{f}}\right)$$

(17) $$i_{t}=\sigma_{\text{th}}\left(W_{i}x_{t}++U_{\text{i}}h_{t-1}+b_{\text{i}}\right)$$

(18) $$o_{t}=\sigma_{\text{th}}\left(W_{\text{o}}x_{t}++U_{\text{o}}h_{t-1}+b_{0}\right)$$

Here, *W*_*i*_, *W*_*o*_, and *W*_*f*_ are the weight matrices of input, output, and forget gates, respectively. Each gate in the LSTM cell is a weight to control how much information can flow through that gate. The input gate controls the flow of values into the cell, the forget gate controls the values that remain in the cell, and the output gate controls the values flowing out of the cell to compute the output activation of the LSTM unit. *U*_*i*_, *U*_*o*_, and *U*_*f*_ are the weight matrices of recurrent connections of input, output, and forget gates, respectively.

(19) $$c_{t}=f_{t}^{\circ }c_{t-1}+i_{t}^{\circ }\sigma_{\text{th}}\left(W_{c}x_{t}+U_{c}h_{t-1}+b_{c}\right)$$

(20) $$h_{t}=o_{t}^{\circ }\sigma_{h}\left(c_{t}\right)$$

**FIGURE 10:**
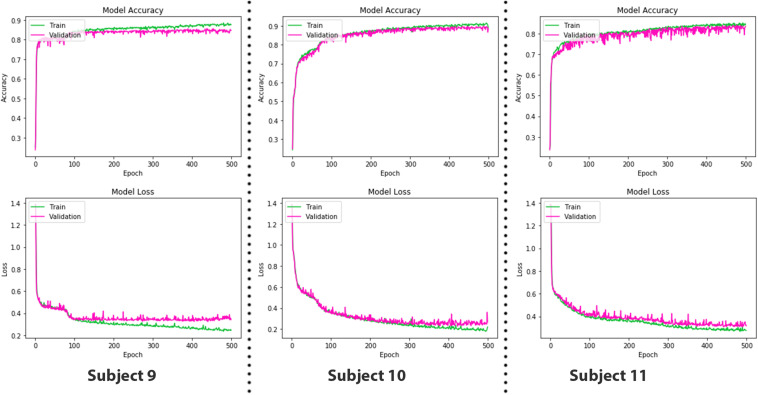
The epoch versus accuracy and loss plots of long short-term memory (LSTM) on train and validation datasets.

As LSTM is being used for time-series data (vector notation), in Eqs 19 and 20, *c*_*t*_ ∈ℝ^*d*^ is not a single LSTM unit but contains *h* LSTM unit cells. σ_th_ is the hyperbolic tangent activation function, and sigmoid function can also be used as an activation function, where *x*_0_, *f*_*t*_, *i*_*t*_, *o*_*t*_, *h*_*t*_, and *c*_*t*_ ∈ℝ^*d*^ and are input vector of the LSTM unit; activation vector of forget gate, input gate, and output gate; output vector of LSTM unit and cell state vector, respectively. *W*ℝ^*h*×*d*^, *U*ℝ^*h*×*h*^ and b  ℝ^*h*^, are the weight matrices and bias vector learned during training. The initial values are *c*_0_ = 0 and *h*_0_ = 0. The operator denotes the element-wise product, and the subscript *t* indexes the time step. In Eqs 18 and 19, it can be seen that output *o*_*t*_ and current state vector *c*_*t*_ at time *t* not only depend on input *i*_*t*_ but are also related to the information at a previous time of LSTM cell. In this manner, LSTM is permitted to remember the important information in the time domain. The superscript *d* and *h* refer to the number of input features and the number of hidden units. In our study, the values of *d* and *h* are 24 and 64, respectively. The complete parameters and layer structure of the proposed LSTM network are shown in Figure 9B. It consists of four LSTM layers, and then the output from the last LSTM layer is flattened and fed into a dense layer that terminates into the final output layer after passing through another fully connected layer. The generalized overview of the implemented LSTM network is presented in Figure 9C. The epoch versus accuracy and loss plots of LSTM on train and validation datasets are shown in Figure 10. For training data, the batch size of 150 is used over 500 epochs for each participant. Accuracies, precision, and recall are presented in section “Results.”

## Results

The results using different classifiers are presented in this section. For all subjects, statistical significance of data per channel is calculated, and only those channels that are employed in classification classifiers are statistically significant. The criteria used for selection of channels are discussed in section “Statistical Significance of Functional Near-Infrared Spectroscopy Data” and Figure 4. For two feature combinations, Signal Mean (M) and Signal Slope (S) produced the best results, which are shown in Figures 5A,B for Subject 1. Average accuracies across 12 channels show that the highest average classification accuracy achieved with SVM and *k*-NN is 54.33 and 54.31%, respectively.

**FIGURE 11:**
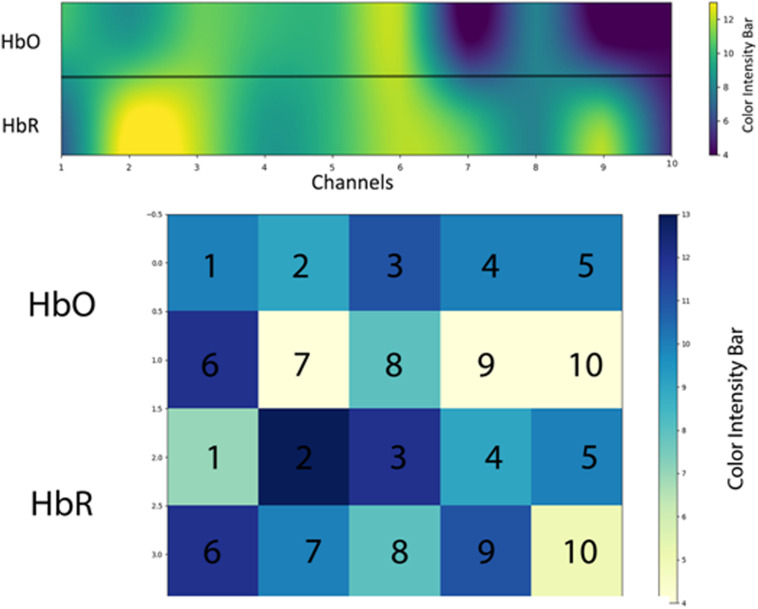
The regions of interest (ROIs) using oxyhemoglobin (HbO) and deoxyhemoglobin (HbR)—response for a specific activity compared with the other areas in prefrontal cortex (PFC). Activation map averaged over all 15 subjects (only significant channels are considered) during activation.

Regions of interest (ROIs) represent the area of the brain that shows the increased response for a specific activity than do other areas in PFC. In this study, ROI is calculated using percentage as a criterion. The studies of Hiroyasu et al. (2015); Hong and Santosa (2016), and Hiwa et al. (2017) are referred as to benchmark studies in measuring ROI; the only difference is that we used a percentage instead of critical *t*-value (*t*_crt_) in calculating ROIs. Different channel positions are highlighted in ROI with varying intensities in an activation map as mentioned in Figure 11. We used SVM accuracies and the color map obtained after setting critical percentage level 55% as shown in Figure 11.

Table 1 entails ANN in comparison with CNN averaged accuracies of 12 channels of each subject. To get a better statistical insight of data, precision and recall are also measured. The precision and recall values are also given alongside the accuracies. The average accuracy of ANN is 69.36% as mentioned in Table 1. Classification accuracies, precision, and recall of all participants calculated using CNN classifier are also summarized in Table 1, with an average accuracy of 87.45%.

**TABLE 1:** Artificial neural network (ANN) and convolutional neural network (CNN) accuracies, precision, and recall of all subjects (in percentage).

	S1			S2			S3		
	**Accuracy**	**Precision**	**Recall**	**Accuracy**	**Precision**	**Recall**	**Accuracy**	**Precision**	**Recall**
ANN	80.66	85.71	81.63	77.66	82.54	77.84	69.91	78.83	70.07
CNN	82.36	87.86	78.75	92.31	94.58	83.15	90.56	92.96	84.50

	**S4**			**S5**			**S6**		
	**Accuracy**	**Precision**	**Recall**	**Accuracy**	**Precision**	**Recall**	**Accuracy**	**Precision**	**Recall**

ANN	68.45	73.57	67.91	55.95	72.71	55.84	78.4	85.79	78.22
CNN	78.24	88.23	85.76	90.66	93.14	86.32	93.02	94.65	86.60

	**S7**			**S8**			**S9**		
	**Accuracy**	**Precision**	**Recall**	**Accuracy**	**Precision**	**Recall**	**Accuracy**	**Precision**	**Recall**

ANN	79.54	83.97	81.88	57.86	74.69	56.72	64.56	76.92	64.47
CNN	86.18	90.30	86.82	85.41	89.76	87.79	86.32	89.86	90.03

	**S10**			**S11**			**S12**		
	**Accuracy**	**Precision**	**Recall**	**Accuracy**	**Precision**	**Recall**	**Accuracy**	**Precision**	**Recall**

ANN	79.29	84.46	78.63	74.74	79.84	74.54	57.6	72.79	57.76
CNN	89.01	91.63	90.25	85.42	89.08	91.71	83.85	90.23	91.93

	**S13**			**S14**			**S15**		
	**Accuracy**	**Precision**	**Recall**	**Accuracy**	**Precision**	**Recall**	**Accuracy**	**Precision**	**Recall**

ANN	68.95	78.28	67.97	61.05	73.54	60.06	65.79	77.52	65.26
CNN	92.54	95.28	92.75	86.78	91.87	93.32	89.13	92.50	93.79

We calculated the classification accurateness of model by the metric “accuracy,” which is the number of correct predictions from all predictions made. To validate the model accuracies and class balance, further model precision (number of positive predictions divided by total number of positive class values predicted) and recall (number of positive predictions divided by the number of positive class values in the test data) are also calculated in Tables 1, 2 to assure class balance in their alignment with accuracy.

**TABLE 2:** Classification accuracies, precision, and recall achieved through proposed long short-term memory (LSTM) network (in percentage).

S1			S2			S3		
Accuracy	Precision	Recall	Accuracy	Precision	Recall	Accuracy	Precision	Recall
83.11	85.34	83.84	89.09	89.53	89.12	87.52	88.68	88.51

**S4**			**S5**			**S6**		
**Accuracy**	**Precision**	**Recall**	**Accuracy**	**Precision**	**Recall**	**Accuracy**	**Precision**	**Recall**
95.51	84.03	83.86	90.85	92.16	91.84	90.42	93.38	92.44
**S7**			**S8**			**S9**		
**Accuracy**	**Precision**	**Recall**	**Accuracy**	**Precision**	**Recall**	**Accuracy**	**Precision**	**Recall**

84.29	88.25	88.37	92.97	86.65	86.40	87.27	85.66	85.49

**S10**			**S11**			**S12**		
**Accuracy**	**Precision**	**Recall**	**Accuracy**	**Precision**	**Recall**	**Accuracy**	**Precision**	**Recall**

95.05	89.76	89.28	84.94	83.68	83.72	84.79	77.88	74.63

**S13**			**S14**			**S15**		
**Accuracy**	**Precision**	**Recall**	**Accuracy**	**Precision**	**Recall**	**Accuracy**	**Precision**	**Recall**

93.40	89.79	88.68	90.77	86.81	85.06	89.78	90.95	90.12

Table 2 presents classification results using LSTM classifier. The highest accuracy achieved with CNN is 93.02%, whereas the highest accuracy with LSTM is 95.51%, which shows that the classification achieved with LSTM has the highest accuracy.

**FIGURE 12:**
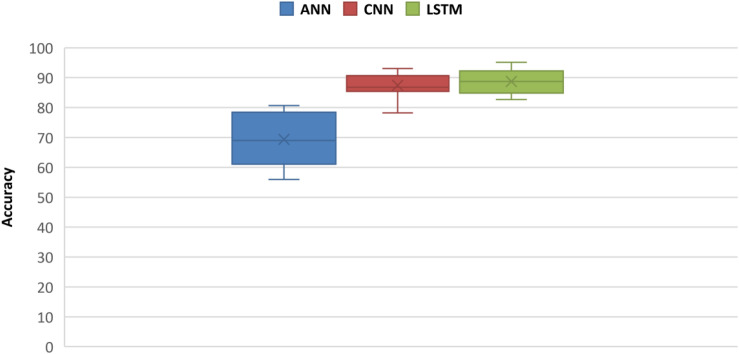
Box plot comparison between ANN, CNN, and LSTM classification accuracies.

Statistical analysis is performed on accuracies obtained through ANNs, CNN, and LSTM. Independent-samples *t*-test was performed between ANN and CNN and between CNN and LSTM accuracies. Results shows that for both statistical tests, *p* < 0.05 and the null hypothesis (with no statistical significance) is rejected. A comparison between ANN, CNN, and LSTM is obtained using one-way *F*-test (ANOVA) to measure inter-similarity between groups (ANN, CNN, and LSTM) on the basis of their mean similarity and *f*-score. Results shows that three groups at a time are also statistically significant with *p* < 0.05. The statistical analysis is coded in software Anaconda IDE with Python 3.7 used with Numpy, and Scikit library, and the software script used to calculate results is added as Annexure C (Supplementary Material). The comparative results between accuracies of ANN, CNN, and LSTM are presented in box plots in Figure 12.

## Discussion

In various brain imaging studies, fNIRS is used to investigate the hemodynamic activities and cognitive states such as MWL, vigilance, fatigue, and stress levels (Cain, 2007; Herff et al., 2013; Ho et al., 2019). Owing to the optical nature of fNIRS, the methodology is less prone to artifacts like a heartbeat or motor, head, and eye movements, which makes it the prevalent choice over other neuroimaging modalities like EEG, PET, and fMRI (Ozge Mercanoglu et al., 2017). The primary aim of this study was to explore the optimal ML or DL algorithms that best fit the four-phase MWL assessment and classification. The cutting edge of DNN over ML is its automatic feature extraction scheme acquired brain signals that override the ML algorithms. In DL, the CNN has a powerful convolutional map to learn classifiable features, and LSTM has memory units to better keep records of time-series patterns, which in our case was the most relevant one. The major takeaway of this study is the application of LSTM for the first time in the classification of a multiobjective task problem.

**FIGURE 13:**
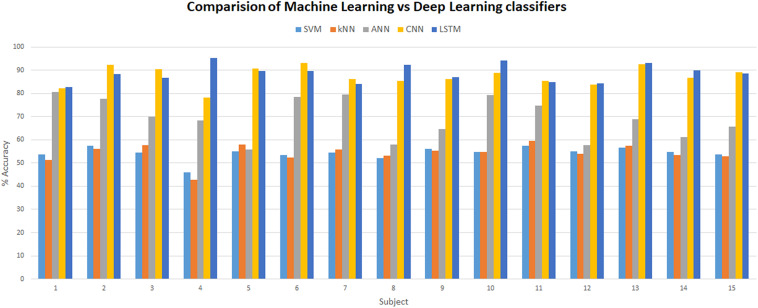
Comparison between machine learning (SVM, *k*-NN, and ANN) and deep learning (CNN and LSTM) classifiers. SVM, support vector machine; *k*-NN, *k*-nearest neighbor; ANN, artificial neural network; CNN, convolutional neural network; LSTM, long short-term memory.

Many fNIRS studies have been carried out to improve classification accuracies of different brain states by using different combinations of features using ML classifiers (Liu and Ayaz, 2018). Best-feature combinations are also shown in various studies, signal slope S (Power and Chau, 2013; Schudlo and Chau, 2013), signal mean M (Faress and Chau, 2013; Naseer and Hong, 2013), signal variance V (Tai and Chau, 2009; Holper and Wolf, 2011), signal kurtosis K (Holper and Wolf, 2011; Naseer et al., 2016), signal skewness SE (Tai and Chau, 2009; Holper and Wolf, 2011), signal peak P (Naseer and Hong, 2015), signal amplitude A (Cui et al., 2010; Stangl et al., 2013), and zero-crossing (Tai and Chau, 2009). Most commonly used features that showed sustainable results are the M, S, and P (Coyle et al., 2004; Fazli et al., 2012; Hong et al., 2015; Khan et al., 2018). In this study, we explored different combinations of two-dimensional (2D) features and concluded M (signal mean) and S (signal slope) combination as the optimal features’ combinations with classification average accuracies of 54.33% (SVM) and 54.31% (*k*-NN), which are in accordance with previous studies and summarized in Figure 13.

Aside from data mining and manual feature engineering, ML classifiers struggle to generalize complex data patterns and, hence, showed poor performance in situations like higher BCI protocols with increased levels of MWL. MWL (using fNIRS) four-phase classification is not very common, and most of the studies are limited to two MWL states, and very few studies explored three MWL phases with conventional ML techniques. Hortal et al. (2013) achieved 87% accuracy on the classification of two mental tasks using SVM. Tai and Chau (2009) reported 96.6 and 94.6% as the highest accuracy of single-trial classification of NIRS signals during emotional induction tasks using LDA and SVM, respectively. Naseer and Hong (2013) reported 87.2% accuracy on two brain signals using SVM. In these studies, as the number of discriminatory phases increases classification, accuracies of ML algorithms decrease. Stangl et al. (2013) classified fNIRS signals during baseline, motor imagery, and MA with an accuracy of 63%. Power et al. (2012) and Yoo et al. (2018) discriminated between three mental tasks with average accuracies of 37.96 and 62.5% using SVM and LDA, respectively. ANN has a higher generalization ability over complex patterns owing to the presence of a huge number of parameters, layers, and non-linear transfer functions. ANN shows better-improved accuracies over other conventional ML techniques. Hennrich et al. (2015) reported 84% accuracy on three mental states using neural network (NN). Abibullaev et al. (2011) managed to get a minimum 71.88% and more accuracy with different NN architectures. In this study, the average ANN accuracy is 69.36%, whereas the highest accuracy with ANN is 80.66%. ANN requires different features, and as the number of features increases, the lgorithms suffer from the curse of dimensionality. The dimensionality of ANN increases as the number of selected features times the number of channels increases, which makes dataset huge and computationally expensive. To cater this “curse of dimensionality,” advance algebraic techniques like principal component analysis (PCA), independent component analysis (ICA), isomap spectral embedding, and QR matrix factorization are used in various studies (Huppert et al., 2009). Also, if data are not carefully preprocessed, over-fitting counterfeits the results on validation set, and algorithms may fail on real-time test data (Cawley and Talbot, 2010).

The trend of employing DNN for classification in BCI and MWL analysis is increasing over the past few years (Nagel and Spüler, 2019). Hennrich et al. (2015) used DNN to effectively classify brain signals. Naseer et al. (2016) analyzed the difference between two cognitive states (MA and rest) on the basis of fNIRS signals using multilayer perceptron (MLP). Huve et al. (2017) classified the fNIRS signals with three mental states including subtractions, word generation, and rest. They employed an MLP model for classification. In another study, Huve et al. (2018) repeated the same procedure for binary classification to control a robot. Hiwa et al. (2016) and Ozge Mercanoglu et al. (2017) attempted to predict the gender of the subjects through their unique fNIRS signals. Saadati et al. (2019a, b) employed CNN using hybrid fNIRS–EEG settings for three-level MWL classification. Ho et al. (2019) developed DBN and CNN for discriminating MWL levels from multichannel fNIRS signals. Left and right motor imageries were classified using DNN in the study of Thanh et al. (2013), and different mental tasks were classified by Abibullaev et al. (2011). In this study, we employed Conv1D CNN architecture, which is a variant of CNN tweaked specifically for time-varying data.

The strength of CNN lies in its self-feature extracting mechanism, which makes it not only powerful but also a preferable choice over the ML algorithms. CNN can independently be used as a full-fledged classifier (feature extraction plus classification) or as a feature extractor with ML classifiers (Tanveer et al., 2019; Zhang et al., 2019). The latter method is to use convolution layers as feature extractors, and acquired features from any fully connected layer are used by ML classifiers like SVM or *k*-NN for classification. This approach has recently been used in fNIRS BCI study (Tanveer et al., 2019) where brain heat maps are used as datasets. In this approach, the training time and computational resources required to train the CNN model increase many folds because time-series data correspond to a single vector, and the images are 2-D and 3-D matrices (2-D in case of gray scale and 3-D in case of RGB image). Matrix manipulation and operations are always expensive in terms of computation than vector operations. The same is true for the forward pass (test time) as well. Our recommendation is to use 1 × 1 bottleneck, and 3 × 3 and 5 × 5 filters for increasing non-linearity and dimensionality reduction in the network instead of using separate classifiers (Lin et al., 2013). In this study, the highest accuracy achieved on any subject with CNN is 93.02%. CNN outperforms all ML algorithms including ANN with a huge margin, as presented in Figure 13. For the verification of experimental paradigm, MWL task difficulty validation is measured with subjective measure NASA-TLX index. In future research, SWAT analysis can also be used to gauge the strength and reliability of an experimental paradigm. Further research could be used to explore the full potential of LSTM in a multitask environment with the application of big data MWL analysis using real-time neuroergonomics and neurofeedback settings.

Long short-term memory is a variant of RNN that uses internal state (memory) to process the sequence of input (Li and Wu, 2015). LSTM shows remarkable improvement in case of time-series data like speech recognition and text-to-speech conversions (Gers and Schmidhuber, 2001; Gers et al., 2003; Graves et al., 2009, 2013; Li and Wu, 2015). So LSTMs are well suited for classifying, processing, and forecasting predictions on the basis of time-series fNIRS data. This is the first study to explore the classification capabilities of LSTM for four MWL phases on time-series fNIRS brain signals. In this study, results showed outstanding performance (highest accuracy) of LSTM over ML classifiers (highest accuracy) and even above DL-CNN (highest accuracy 93.02%). LSTM outperformed the current state-of-the-art algorithm on CNN by more than 2.51%. The highest accuracy achieved with LSTM is 95.51%. Figure 13 shows a detailed comparison of DL (LSTM and CNN) and ML (ANN, SVM, and *k*-NN) classifiers. Being a relatively new algorithm (LSTM) in neuroscience, there is a lot of room for further research and exploration. Computational time and resources required for LSTM and other ML and DL classifiers can also be compared and analyzed in future research studies.

## Conclusion

In this study, four-state MWLs were evaluated and classified using three ML (SVM, *k*-NN, and ANN) and two DL (CNN and LSTM) algorithms using fNIRS hemodynamics signals. Data reliability and significance are validated by *p*- and *t*-tests per channel. Nine extracted features from original hemodynamic signals were used with two feature combination arrangements for ML classification. The signal mean–slope (M–S) combination yielded the average classification accuracy of 54.33, 54.31, and 69.36% using SVM, *k*-NN, and ANN, respectively. Averaged classification accuracy achieved by CNN is 87.45%, and it outperformed all conventional classifiers by an acceptable margin. This study shows that LSTM can be effectively used for optimum classification of MWL-fNIRS brain signals with classification accuracies ranging from 83.11 to 95.51%. Classification accuracies of LSTM are compared with the accuracies achieved using SVM, ANN, KNN, and CNN methods. LSTM works better than CNN, ANN, and other conventional classifiers. The average accuracy achieved with LSTM is 89.31%, which is greater as compared with the average accuracy (87.45%) acquired using CNN. The novelties of this study are that four levels of MWL are estimated using a combination of mental logic and MA tasks and also for the first time LSTM is implemented on four-level MWL-fNIRS data with achieved optimum classification accuracies.

## Data Availability Statement

All datasets generated for this study are included in the article/Supplementary Material.

## Ethics Statement

The studies involving human participants were reviewed and approved by the Ethical Research Council of RISE at SMME—National University of Sciences and Technology (NUST). The patients/participants provided their written informed consent to participate in this study.

## Author Contributions

UA, KK, and NN contributed to the conception of the study. UA and MK provided the methodology. UA, KK, and MK acquired the software, conducted the investigation, and formally analyzed the results. MK, NN, and UA validated the results. RA, YA, and SB acquired the resources and supervised the study. UA, KK, and YA wrote the original draft. UA, MK, SB, and KK revised and edited the manuscript. UA, NN, and SN provided the visualization. RA, SB, YA, and SN administered the experimentation. All authors contributed to the article and approved the submitted version.

## Conflict of Interest

The authors declare that the research was conducted in the absence of any commercial or financial relationships that could be construed as a potential conflict of interest.
