# Supplementary material for: Enhanced Accuracy for Multiclass Mental Workload Detection Using Long Short-Term Memory for Brain–Computer Interface
Source: Front Neurosci. 2020 Jun 23;14:584. doi: 10.3389/fnins.2020.00584 (PMC7324788; doi:10.3389/fnins.2020.00584)
Supplement: Supplementary file 1 [file Data_Sheet_1.PDF]

# Raw data of Participant "ByAO1HXP8" in Experiment "2"

Experiment performed at April 2nd 2020, 3:35:08 pm

## Weighted rating: 52

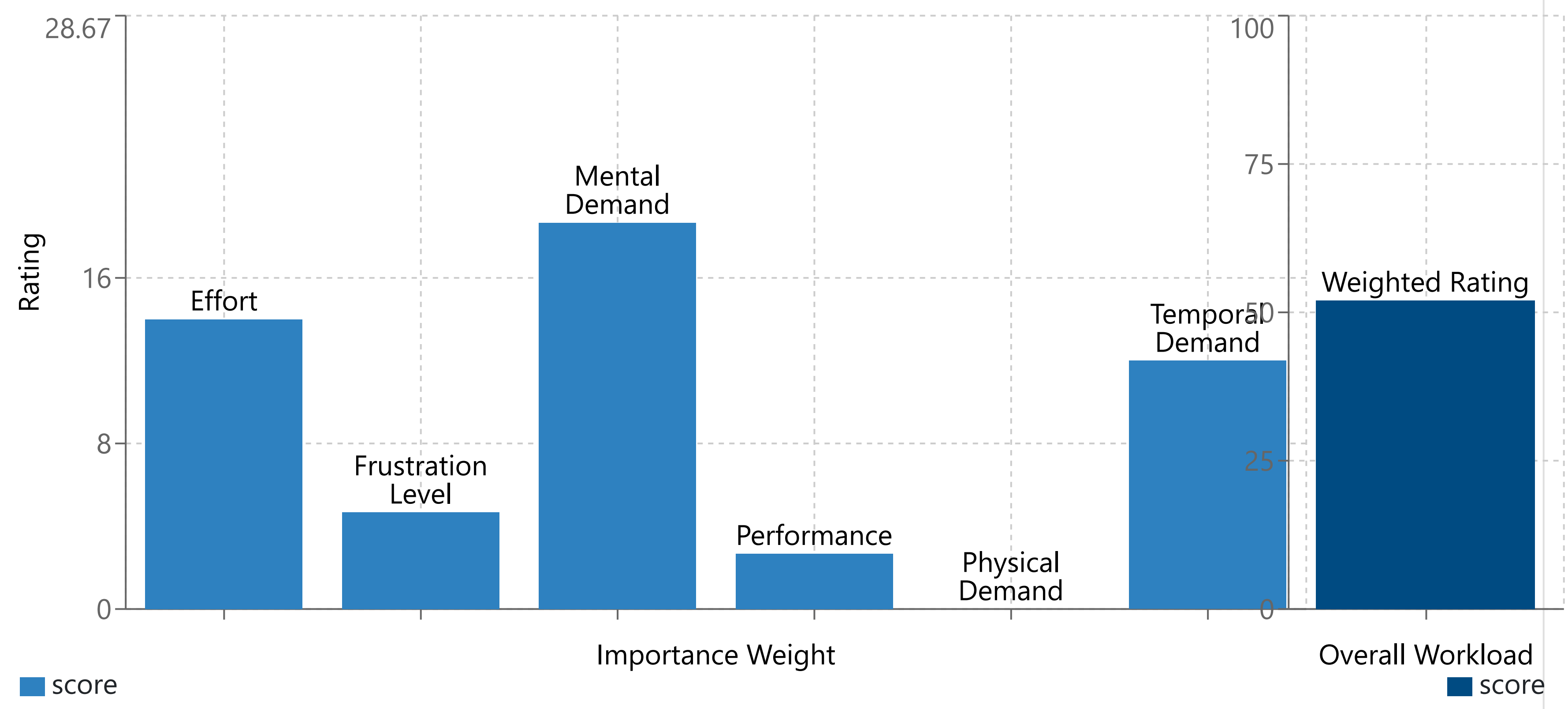


### Raw ratings

| Effort | Frustration Level | Mental Demand | Performance | Physical Demand | Temporal Demand |
|---|---|---|---|---|---|
| 70 | 70 | 70 | 10 | 20 | 60 |

### Sources of Workload tally (number of times selected)

| Effort | Frustration Level | Mental Demand | Performance | Physical Demand | Temporal Demand |
|---|---|---|---|---|---|
| 3 | 1 | 4 | 4 | 0 | 3 |

### Adjusted Rating (Weight x Raw)

| Effort | Frustration Level | Mental Demand | Performance | Physical Demand | Temporal Demand |
|---|---|---|---|---|---|
| 210 | 70 | 280 | 40 | 0 | 180 |

# Raw data of Participant "rye_WHQPU" in Experiment "3"

Experiment performed at April 2nd 2020, 3:43:48 pm

## Weighted rating: 66

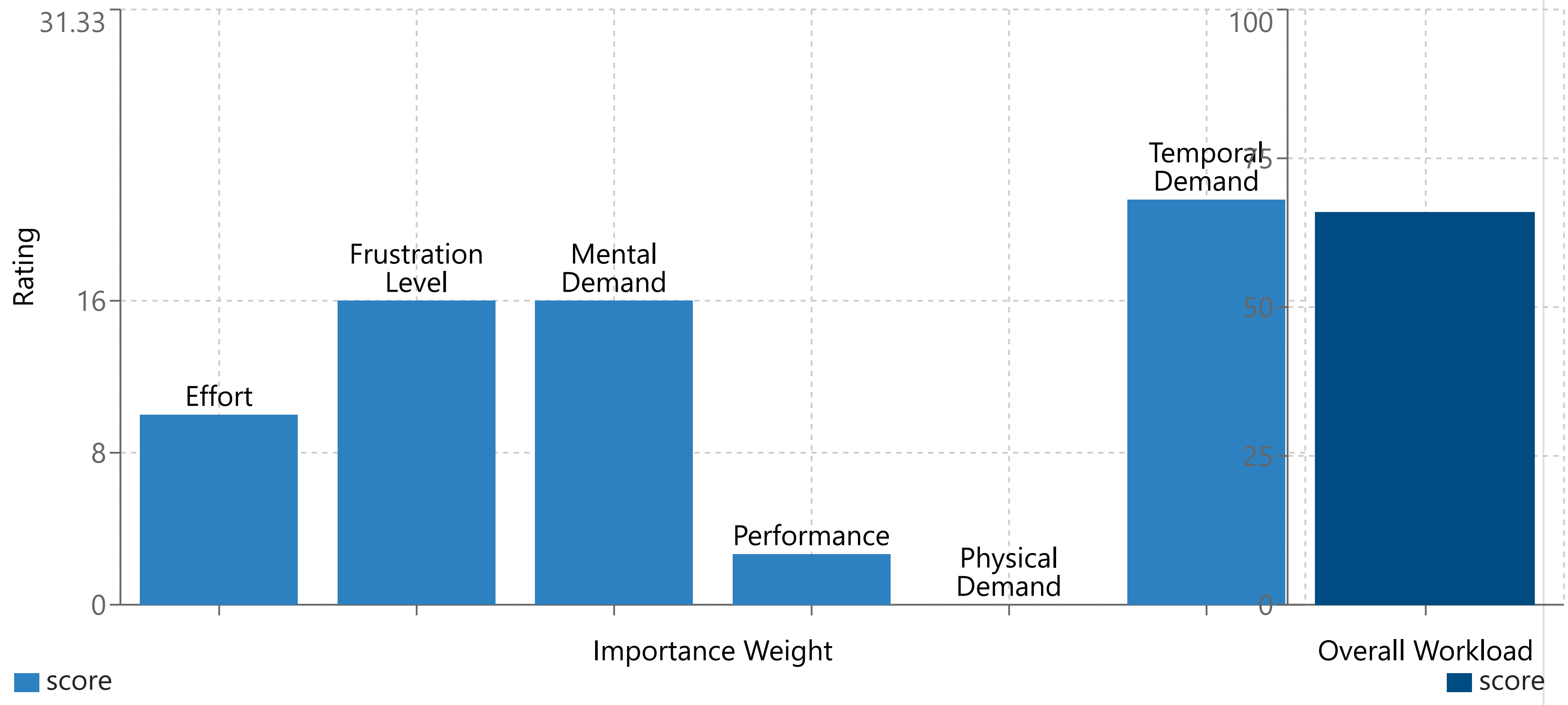


### Raw ratings

| Effort | Frustration Level | Mental Demand | Performance | Physical Demand | Temporal Demand |
|---|---|---|---|---|---|
| 50 | 80 | 80 | 20 | 50 | 80 |

### Sources of Workload tally (number of times selected)

| Effort | Frustration Level | Mental Demand | Performance | Physical Demand | Temporal Demand |
|---|---|---|---|---|---|
| 3 | 3 | 3 | 2 | 0 | 4 |

### Adjusted Rating (Weight x Raw)

| Effort | Frustration Level | Mental Demand | Performance | Physical Demand | Temporal Demand |
|---|---|---|---|---|---|
| 150 | 240 | 240 | 40 | 0 | 320 |

# Raw data of Participant "HJtNMB7DL" in Experiment "4"

Experiment performed at April 2nd 2020, 3:46:39 pm

## Weighted rating: 80.67

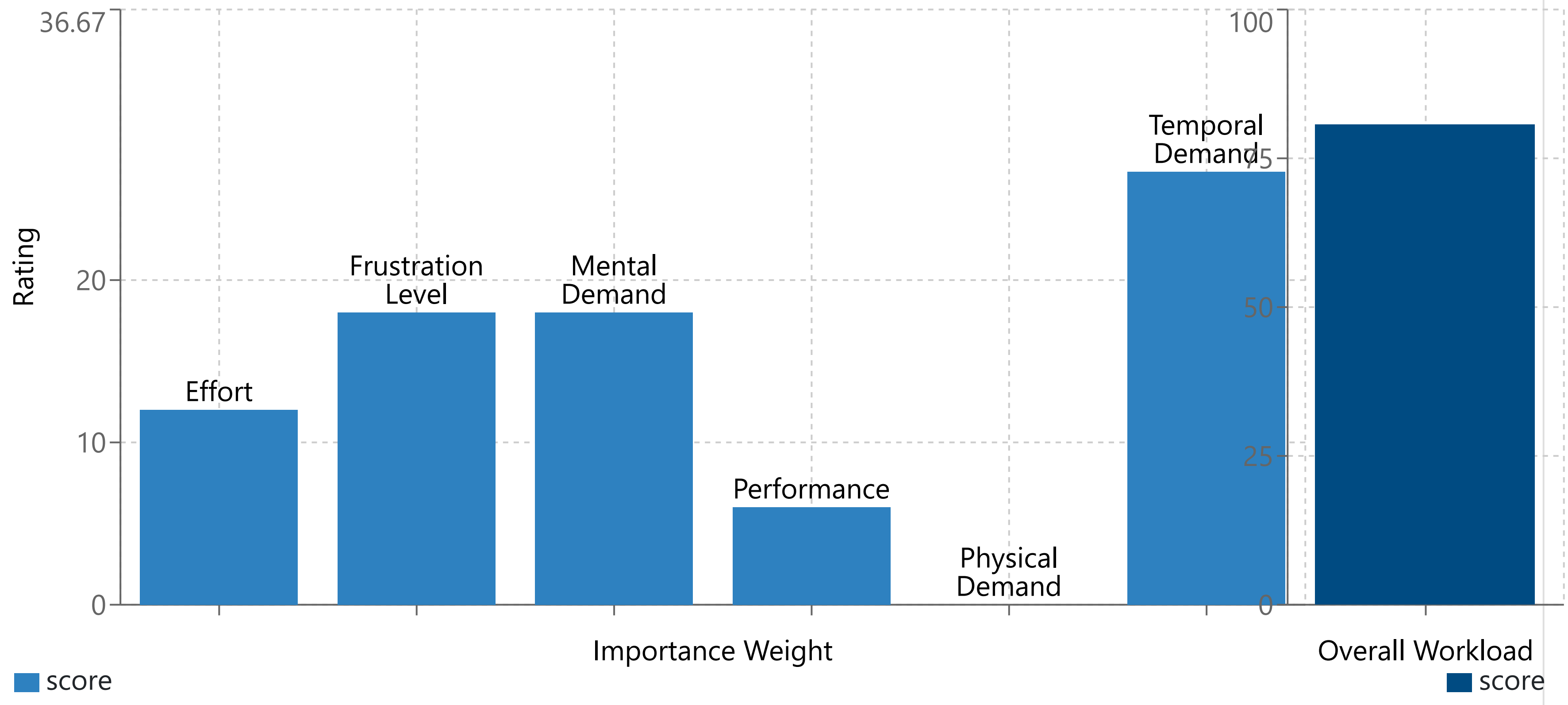


### Raw ratings

| Effort | Frustration Level | Mental Demand | Performance | Physical Demand | Temporal Demand |
|---|---|---|---|---|---|
| 90 | 90 | 90 | 30 | 50 | 100 |

### Sources of Workload tally (number of times selected)

| Effort | Frustration Level | Mental Demand | Performance | Physical Demand | Temporal Demand |
|---|---|---|---|---|---|
| 2 | 3 | 3 | 3 | 0 | 4 |

### Adjusted Rating (Weight x Raw)

| Effort | Frustration Level | Mental Demand | Performance | Physical Demand | Temporal Demand |
|---|---|---|---|---|---|
| 180 | 270 | 270 | 90 | 0 | 400 |
