# Supplementary material for: Enhanced Accuracy for Multiclass Mental Workload Detection Using Long Short-Term Memory for Brain–Computer Interface
Source: Front Neurosci. 2020 Jun 23;14:584. doi: 10.3389/fnins.2020.00584 (PMC7324788; doi:10.3389/fnins.2020.00584)
Supplement: Supplementary file 2 [file Data_Sheet_2.PDF]

Time Series averaging of MWL states signals:

Time series signals plots (MWLs) are created by using fNIRS time series data and initial pre-processing (filtering, noise removal) is applied on data. From each subject's data, only activity periods of HbO channels for MWL 1, 2, 3, and baseline state is retained, rest is discarded. The data is spatially averaged across all channels (HbO), as a result, only a single column vector is obtained that contains averaged effect of all channels. This column vector is then broken as per the number of trials of each activity. The number of trials (10 in our case) are then temporally averaged for each activity. This results in 4 column vectors. One for each measured hemodynamic response (HR) of mental workload state. Then polynomial curve fitting from Scipy library used and fitted on the measured averaged HR. Figure shows Time vs amplitude plot of spatially averaged HR response using spatially averaged HbO. Data contains the averaged information of 10 trials and each trial spawns for 20 seconds.

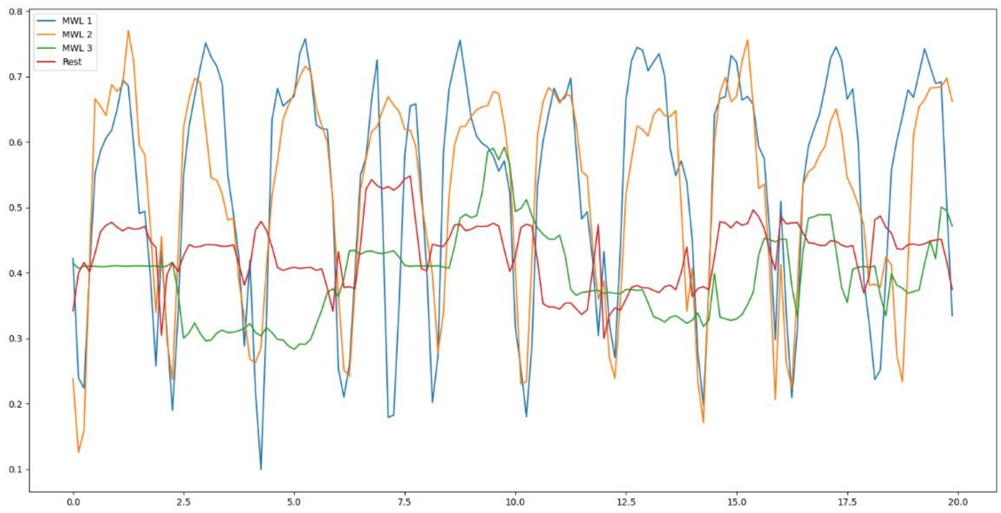

Figure: The plot of spatially averaged HR responses with varying levels of MWLs
