# Supplementary material for: Enhanced Accuracy for Multiclass Mental Workload Detection Using Long Short-Term Memory for Brain–Computer Interface
Source: Front Neurosci. 2020 Jun 23;14:584. doi: 10.3389/fnins.2020.00584 (PMC7324788; doi:10.3389/fnins.2020.00584)
Supplement: Supplementary file 3 [file Data_Sheet_3.PDF]

```
# -*- coding: utf-8 -*-
"""
Created on Mon Mar 16 14:15:55 2020

@author: MSI
"""
import pandas as pd
import matplotlib.pyplot as plt
import numpy as np
from scipy.stats import ttest_ind
from scipy import stats

ml_acc = 
[76.04,88.02,85,71.09,85.83,86.3,82.13,85,75.67,84.47,82.23,66.25,88.38,82.03
,88.48]
cnn_acc = 
[82.76041667,93.125,91.40625,78.59375,91.71875,93.64583333,86.40625,86.145833
33,86.5625,89.84375,85.52083333,84.32291667,92.65625,87.65625,90.20833333]
lstm_acc = 
[82.70833333,88.28125,86.66666667,95.15625,89.79166667,89.79166667,84.0625,92
.23958333,87.03125,94.21875,84.84375,84.32291667,93.28125,89.89583333,88.6979
1667]

ttest1, pval1 = ttest_ind(ml_acc, cnn_acc)

print("p-value = ", pval1)
if pval1 < 0.05:
    print("we REJECT null hypothesis for ML, DL accuracies being random")
else:
    print("we ACCEPT null hypothesis for ML, DL accuracies being random")

ttest2, pval2 = ttest_ind(cnn_acc, lstm_acc)

print("p-value = ", pval1)
if pval2 < 0.05:
```

```
    print("we REJECT null hypothesis for DL, LSTM accuracies being random")
else:
    print("we ACCEPT null hypothesis for DL, LSTM accuracies being random")


''' ONE WAY ANOVA test for all groups at once'''


F, p = stats.f_oneway(ml_acc, cnn_acc, lstm_acc)


print("p-value = ", p)
if p < 0.05:
    print("we REJECT null hypothesis for Ml, DL and LSTM accuracies being 
random")
else:
    print("we ACCEPT null hypothesis for ML, DL and LSTM accuracies being 
random")
```
